# Validity evidence for the coping strategy indicator-short version (CSI-S) among psychology students

**DOI:** 10.1038/s41598-024-55659-5

**Published:** 2024-03-05

**Authors:** César Merino-Soto, José Livia-Segovia, Marivel Aguirre-Morales, Filiberto Toledano-Toledano

**Affiliations:** 1 Instituto de Investigación en Psicología, Universidad de San Martin de Porres, Surquillo, Peru; 2 Universidad Nacional Federico Villareal, Lima, Peru; 3 Unidad de Investigación en Medicina Basada en Evidencias, Hospital Infantil de México Federico Gómez Instituto Nacional de Salud, Mexico City, Mexico; 4 Unidad de Investigación Multidisciplinaria en Salud, Instituto Nacional de Rehabilitación Luis Guillermo Ibarra Ibarra, Mexico City, Mexico; 5 Dirección de Investigación y Diseminación del Conocimiento, Instituto Nacional de Ciencias e Innovación para la Formación de Comunidad Científica, INDEHUS, Mexico City, Mexico

**Keywords:** Psychology, Environmental social sciences

## Abstract

The abbreviated measurement of coping strategies is useful for monitoring and identifying the effects of stress. The Coping strategy indicator-Short version (CSI-S, including the dimensions of seeking support, problem solving and avoidance strategies) is a new adaptation of the full version of this indicator, and additional evidence of its validity is needed. Psychology students (n = 125) from a public university in Lima, Peru, were recruited to help provide such evidence of validity in terms of internal structure, reliability and associations with other variables (perceived stress and general efficacy in cope with difficulties), which were evaluated using nonparametric item response theory procedures. Support-seeking and problem-solving items from the Mokken scale and the avoidance scale exhibited limitations. The correlations between the scales were moderate or low and exhibited theoretical consistency, and the relationship with perceived stress highlighted the predictive capacity of avoidance and problem-solving strategies. In general, the CSI-S exhibits suitable psychometric properties; however, the avoidance score requires further examination or reconstruction of its items.

## Introduction

Among workers, stress is linked with measures of well-being, psychological symptoms and sleep disturbances^1,2^. It is known that increased stress covaries monotonically with decreased performance and motivation alongside an increased risk of discontinuing education in the university students population^3^. This impact appears to lead to a variety of additional problems, such as symptoms of anxiety, depression, and negative affect as well as a general state of deteriorating positive expectations about the value of education^3,4^. The effects of these emotional and social-cognitive symptoms can be directed toward the student's academic performance and engagement in their studies^4^ and can be viewed as a network of factors and consequences linked to stress. In this sense, without the implementation of effective stress coping strategies, the deterioration in mental health and academic efficacy and efficiency would increase in young adults.

For this reason, the implementation of effective coping strategies is important for student academic survival and for identifying a protective organizational climate that interacts with the potential efficacy of individual coping^3^. However, coping strategies do not function identically; for example, avoidant-type strategies are often ineffective at reducing the effects of stress^5,6^, while problem-solving strategies decrease its occurrence^7^.

The complex study of coping strategies involves an extensive map of coping behaviors operationalized by different instruments that can be applied to general daily life or specific situations^8^. Some patterns of coping responses seem to be consistent around the world. For example, in a recently published systematic literature review from Asia, problem-focused strategies were one of the most common coping strategies exhibited by workers in response to work-related stressors^2^; moreover, this same type of coping and coping based on social support seeking seem to be common in other groups^9^.

A recent abbreviated measure of coping strategies is the Coping strategy indicator–Short version (CSI-S^10^), which describes three general coping behaviors: social support seeking, problem solving and avoidance. The CSI-S is structured similarly to the full Anglo-Saxon version^11^ with regard to the number of coping strategy dimensions it assesses, the symmetrical distribution of items in each dimension, and the number of response options. The CSI-S is derived from the CSI, a measure of situational coping, because it associates responses to items with references to specific stressors that the respondent previously identified. The CSI emerged in the context of the Ways of Coping Checklist (WCC^12^), a standard measure of stress coping that had been employed for several years, but the literature has recognized consistent problems with regard to the validity of its internal structure^11,13–15^. These problems highlight important limitations pertaining to construct definitions based on item content and uneven descriptions of these constructs across different groups and contexts^16–25^.

The original 33-item version (CSI^11^) was constructed on an empirical basis, in which context successive analyses of latent variables were conducted to select items that exhibited high stability and internal validity. The three dimensions of the CSI (problem solving [PS], seeking social support [SS], and avoidance [AVO]; 11 items in each) were consistent sources of variability in coping strategies according to Amirkhan, not only because of the robustness of their internal structure but also because of the consistent theoretical convergence of their associations with other variables, such as extraversion^26^, causal attributionss^27^ and sense of coherence beliefs^28^. Additionally, early studies of the CSI highlighted its variability due to demographic factors, such as gender, income, and education^29^. Studies that have used the CSI to investigate homogeneous samples with the CSI^30^, even in the context of investigations of psychology students with sample sizes of approximately 100^31^, as in the present study, have exhibited theoretical convergence with the internal structure of the CSI and its associations with the relevant variables. These three dimensions can be viewed as more prominent latent constructs associated with coping strategies, and the literature has consistently recognized and demonstrated the distinctions among them. That is, other models of coping strategies have identified these three dimensions^32–34^ or included them in a larger set of dimensions^35,36^.

On the other hand, the short version of the CSI-S, which was created by Merino-Soto and Juaréz-García^10^, was developed on a similar theoretical basis but by reference to a heterogeneous sample of young adults in university. Employing an etic conceptualization of the scale (in the sense of the cross-cultural and intercontextual capacity of the constructs^37,38^), the investigation of this new brief version verified that the structure of coping responses can be clearly identified using a certain set of items (including 5 items per dimension). Other brief measures of stress coping have also been used to increase measurement efficiency and decrease internal structure variability: the Coping Inventory for Stressful Situations (CISS^32,39^); the Coping strategy inventory–Short form (CSI-SF^36^); and the Brief Coping Orientation to Problems Experienced Inventory (BRIEF-COPE^33^).

Like the CSI-S and its parent version, the CSI, other measures have been used to map coping to the constructs measured by the CSI-S. For example, brief measures derived from the WCC model have exhibited theoretical overlap, such as the problem-focused, emotion-focused, and seeking social support scales^33,34^. However, problem solving coping can also be conceptualized in terms of task-oriented coping, as in the CISS model^32,39^. On the other hand, the Coping Strategy Inventory^35^ also contains scales that have been directly linked to SP, SS and AVO.

In contrast to the use of the full 33-item version (CSI) in the English-speaking world, the CSI has been studied psychometrically by several studies conducted in Spain^40^ and Latin America^41,42^, and its incorporation into research and professional practice in the Hispanic context seems to require more attention. Due to its parsimonious representation of coping distributed across three constructs, the CSI tends to be robust across its use in different cultural contexts^8^.

Due to its brevity, parsimonious structure, and theoretical coherence, the new version, CSI-S, may be indicated to explore the mediating effect of coping strategies between stressors and stress when time is an issue, as well as to screen the prevalence of coping strategies in daily life, academic, clinical, and research situations. Although no additional validity evidence has been reported regarding the CSI-S and although its equivalence with the full version has been shown to be satisfactory^10^, its usefulness for research is still being confirmed, especially in the university context, where intense experiences of stress accompanied by emotional symptoms, impaired academic performance, and interpersonal relationships are common^3,4^.

To date, little evidence regarding the validity of the CSI-S in university students’ populations has been reported, specifically regarding psychology students, and the stability of its psychometric properties with respect to samples beyond a single initial validation study^10^ remains unknown. Therefore, the present study aims to add evidence of the valid scaling of their scores on the three coping strategies, as well as their correlates with estimated measures of stress and perceived efficacy in the face of difficulties. In this study, partial advances were focused on the exploration of nonparametric procedures, which are efficient, more accurate, and capable of reducing bias in the internal validity of the study due to noncompliance with the assumptions of parametric methods (e.g., normality of the latent attribute and a large sample size^43^). These results complement the rigorous study of internal structure reported by Merino-Soto and Juárez-García^10^ based on a new methodological approach, which is potentially efficient in this small-sample context. However, while the CSI-S first study was subjected to a process of item reduction based on a detailed analysis^10^, other sources of validity were not explored. One of these sources, i.e., relationships with external variables, is required to assess the applied value and theoretical foundation of this approach^29,44–46^. Therefore, the present study explored the validity of the CSI-S with respect to other variables, such as stress and efficacy to cope with difficulties. With respect to these two constructs, (a) in the case of stress, we expected to observe a pattern of positive association with avoidance and a pattern of negative association with problem solving and seeking social support; (b) in the context of coping in difficulties, we expected to observe the opposing pattern, i.e., a pattern of negative association with avoidance and positive association with problem solving and seeking social support.

Overall, these measures were used in the context of potential real-world, nonexperimental application: the evaluation of students in their final years of study. This study contributes to the emerging body of research on the use of the CSI-S to investigate Spanish-speaking participants, which was initiated by Merino-Soto and Juarez-Garcia^10^; in addition, the relevant relationships with other variables have been tested^47^. Methodologically, an ancillary contribution of the study pertains to the use of nonparametric item response theory methodology to maximize the internal validity of the study in a challenging situation in terms of sample size and variability. More precisely, due to the small size of the study and the exploratory objective of the participant group, Mokken scaling analysis (MSA) may be a reasonable choice^48,49^.

## Results

### Preliminary analysis: potential response bias

#### Guttman errors

Through the *G*^+^ score for SS (Md = 0.0, Min = 0.0, Q3 = 3, Max = 12), PS (Md = 0.0, Min = 0.0, Q3 = 2, Max = 11) and AVO (Md = 2.0, Min = 0.0, Q3 = 3, Max = 11) dimensions, five subjects with the most extreme *G*^+^ values were detected; although the distance to the median did not seem to be large, they were still removed from the data. With the updated data on 120 participants, the dimensionality and the relationships of the CSI-S with the criterion variables were analyzed, and the optimal scores were estimated using Ramsay approach.

### Evidence of internal structure

#### Dimensionality

For all items, strong multidimensionality (DETECT = 5.59) and slightly complex structure (ASSI = 0.71, RATIO = 0.87) were found. An exploratory solution evaluating 2 to 4 dimensions verified that the optimal number of scales is 3 (DETECT = 6.63, ASSI = 0.58, RATIO = 0.79), with the items placed in their respective expected scales but with associations between them.

#### Mokken scaling analysis (MSA)

In general, adequate scaling was obtained at the item level and in total SS and PS scores (Table 1), except in AVO (item 6, H < 0.30), which showed moderate scaling. However, the monotonic homogeneity model (crit = 0) was completely satisfactory. No local scale dependence was detected, resulting in low W^(2)^ values for all scales (in Table 1, W^(2)^). Item-test correlations were high on SS (Min = 0.48, Max = 0.69) and PS (Min = 0.58, Max = 0.67), and varied in the low to high range on AVO (Min = 0.24. Max = 0.61). Polyserial correlations were high on all three dimensions (≥ 0.50).

**Table 1:** Nonparametric modeling for the internal structure of the CSI-S (n = 120).

	M	Mokken scaling analysis (MSA)					Classic theory test		
		H	se	CRIT (MHM)	W^(2)^	MS	α	r_itc_	r_pit_
Social support (SS)									
csi1	2.66	0.43	0.09	0	6.66	0.79	0.79	0.48	0.65
csi3	2.60	0.54	0.06	0	4.47			0.62	0.77
csi10	2.18	0.56	0.07	0	4.79			0.54	0.75
csi14	2.58	0.59	0.07	0	4.27			0.69	0.81
csi15	2.62	0.48	0.07	0	6.05			0.55	0.72
Total		0.52	0.06						
Problem solving (PS)									
csi4	2.72	0.52	0.08	0	6.13	0.84	0.83	0.58	0.73
csi5	2.70	0.58	0.06	0	4.99			0.67	0.79
csi7	2.65	0.52	0.07	0	5.73			0.61	0.77
csi9	2.63	0.56	0.07	0	4.30			0.66	0.80
csi12	2.66	0.52	0.07	0	5.83			0.61	0.76
Total		0.54	0.06						
Avoidance (AVO)									
csi2	2.01	0.47	0.05	0	6.57	0.69	0.69	0.55	0.72
csi6	1.58	0.23	0.08	0	10.63			0.24	0.50
csi8	1.77	0.43	0.05	0	7.62			0.54	0.73
csi11	1.92	0.31	0.07	0	10.69			0.36	0.62
csi13	1.79	0.46	0.05	0	5.51			0.61	0.78
Total		0.38	0.04						

*M* mean response, *H* scaling coefficient, *se* standard error of H, *MSA* Mokken scaling analysis, *MHM* monotonic homogeneity model, *CRIT* weighted criterion of fit to the MHM model, *r*_*pit*_ polychoric item-test correlation, *r*_*itc*_ item-test correlation, *MS, α* internal consistency reliability coefficient.

#### Reliability

The MS and α reliability coefficients of the BUAP and SOPRO scores (Table 1) were similar and greater than 0.78, with the exception of the coefficient for EVI, which was comparatively low but greater than 0.65.

### Evidence of associations with external variables

#### Relationships among coping strategies

The linear correlations between the subscales (Table 2) were between trivial and large and were all statistically significant, indicating that they were active in the face of stressors, with different degrees of association.

**Table 2:** Ordinal logistic regression and correlations between the CSI-S score and criterion variables (n = 120).

Predictors	Lineal correlations (95% confidence interval)			Ordinal logistic regression			
				Stress ($R_{MF}^{2}$ = 0.02)		Efficacy to cope ($R_{MF}^{2}$ = 0.05)	
	SS	PS	AVO	Coefficients (se)	OR (IC 95%)	Coefficients (se)	OR (IC 95%)
SS	1			0.109 (0.08)	1.12 (0.94, 1.34)	− 0.045 (0.09)	0.955 (0.795, 1.148)
PS	0.57** (0.43, 0.67)	1		− 0.144 (0.10)	0.86 (0.69, 1.06)	0.325** (0.11)	1.385 (1.108, 1.738)
AVO	− 0.17* (− 0.34, − 0.00)	− 0.39** (− 0.52, − 0.23)	1	0.173* (0.07)	1.17 (1.01, 1.37)	− 0.132 (0.08)	0.875 (0.742, 1.028)

$R_{MF}^{2}$ pseudo R McFadden, *OR* odds ratio, *SS* support seeking score, *PS* problem-solving score, *AVO* avoidance score, *se* standard error.

**p < 0.01, *p < 0.05.

#### Relationships with external variables

Sex and age did not produce statistical influence (McFadden *R*^2^ between 0.002 and 0.006), so they were not included in the final ordinal regression. Table 2 shows the parameters obtained from the analysis. A deviation was observed in the SIS responses with respect to the uniform distribution (Cressie-Read *χ*^*2*^ = 9.839, *p* = 0.02), but it was small (*V*_*Cramer*_ = 0.160^50^); therefore, the logit link function was used. The statistical influence of avoidant coping strategies on the perception of stress and that of focusing on problem solving on the increase in coping efficacy was observed.

## Discussion

The present study obtained results to extend the validity evidence of the CSI-S, a new abbreviated measure of coping strategies. Derived from the full version (i.e., the CSI, 33 items^11^) and based on the study in which it was developed^10^, the results were satisfactory in terms of all of the validity evidence obtained.

Regarding dimensionality, the strongest dimensions were PS and SS, with similar scalability and item-scale association, while AVO was weak in these aspects; the reliability of its scores also maintained this same trend. These results do not differ from the trend observed with regard to the full version^7,11,40–42^ and suggest the conceptual complexity of the AVO construct, which is also reflected in its observed score. Therefore, the weakness of the internal structure of avoidant coping strategies is linked to their conceptually complex nature rather than to problems in their construction. Two implications can be identified: first, the measurement of avoidant coping strategies may require additional assessment to describe it accurately, such as using the full 11-item version (avoidance subscale, in the full CSI^11,42^); additionally, the reliability results thus obtained suggest that group description and screening purposes represent its primary form of use.

Linear covariation among coping strategies suggests moderate dependence among some, for example, between support seeking and problem solving and avoidance and problem solving. One implication is that coping involves not merely the perceived effectiveness of a single predominant strategy or behavior but also the activation of several strategies that synergize to facilitate coping with stressors^5,6,11^. The reported correlations were either low or moderate, which may corroborate the findings reported in the literature regarding the flexibility of coping strategy use. This flexibility may be a predictor of coping in the face of stressful events and has implications for reducing depressive symptoms^51^.

In the assessment of predictive relationships with stress, avoidance (AVO) was the strategy that was most closely linked to an increase in perceived stress, whereas support seeking (SS score) and problem solving (PS score) did not overcome sampling error. This finding confirms theoretical expectations concerning their role in perceived stress in the general population^5,6^ and in the young adult student sample of the present study. In relation to efficacy in coping with difficulties, problem-solving strategies play an incremental role, and both are usually effective at attenuating the effects of stress^5,6^ and producing adaptive responses. On the other hand, avoidance of stressful situations is linked to stress, which replicates the findings of previous studies^3,5,6,11^. The These strategies, especially those that focus on directly managing stressors and active social support seeking, have been shown to be habitual means of effective coping and to lead to better effects on individual well-being^2,9^. Some evidence has shown associations close to zero with respect to the role of problem-based coping^52^; however, these findings may also indicate that some nonlinear associations were not detected.

Some limitations are listed. First, the representativeness of the sample was not assured due to the nonrandom sampling strategy, apparent sample homogeneity, and sample size, thus limiting the statistical stability of the results. Second, although single-item measures are efficient for representing constructs of individual and psychosocial variability, they show reduced reliability and construct coverage, thus replication of the study with multi-item measures is needed. Third, other conceptual links with strong implications for clinical practice and score validation were not included, such as past trauma^5^; accordingly, future research should explore other exposures or stressors. Fourth, the internal structure requires linear parametric modeling, using structural equations, to align with the original study in obtaining psychometric parameters. Finally, the association with the complete version of the CSI was not evaluated, and although the original study^10^ reported satisfactory concurrent correlations, the reproducibility of the equivalence between the two versions has yet to be evaluated.

These limitations must be weighed against other aspects of the study. First, sample homogeneity is not necessarily an issue as long as we do not claim that our results are generalizable. Therefore, given that convenience sampling and small sample sizes are not uncommon in social science and clinical significance measurement studies^53^, this issue should not reduce the substantive significance of the results of this study because the pattern of correlations with external variables and the internal structure replicate the source study for this abbreviated version as well as the findings obtained using the full version. Examples of studies that have measured coping responses by referenced to homogeneous samples can be found in the context of highly specific groups in terms of both health conditions^14,30^ and normal conditions^31^. Therefore, the apparent problem pertaining to the sample size of this research can be viewed in relative rather than absolute terms. In line with the purpose of this study, the relevant research gap, and the context of instrument use, a small sample size may be sufficient or justifiable^54^.

Second, we do not doubt that single-item measures that have rarely been used in Spanish-speaking countries require evidence of validity; however, on this occasion, we rely on previously available international evidence. Thus, single-item measures have been shown to be efficient as well as effective at parsimoniously representing constructs in the context of stress. Additionally, these measures have been recognized for their satisfactory content validity and associations with other variables^47,55,56^.

Our study concludes that the properties of the CSI-S are essentially generalizable in relatively small-sample contexts and that it replicates the metric quality trend of studies conducted using the full version and of the study from which it originated^10^. Additionally, evidence obtained with perceived stress indicates that, in the context of parsimonious measurement with brief or ultrashort measures (e.g.^1,56^), the CSI-S can be useful for screening and monitoring levels of coping strategies. Finally, due to the replicable internal structure of the CSI and CSI-S and the fact that the content is built with items used in different adaptations of the CSI around the world, we think that this version may be useful not only for the specific context of its development (i.e., Peru) but also for other Latin American and Anglo-Saxon groups.

## Methods

### Participants

The study population was psychology students from a Peruvian public university located in Metropolitan Lima who were predominantly of lower middle or low socioeconomic level. All had Spanish as their first language. The nonrandomly accessible sample was students in their last two semesters of study, corresponding to the internship or preprofessional practice period in various Peruvian institutions. The participants in this group were chosen due to the evaluation needs of this group, which feature a high demand for activities, and because stress and coping among this group have been infrequently explored in Peru. There were 125 students, with characteristics presented in Table 3, aged between 23 and 28 years, all born in Lima; the internship sites were distributed over a wide range of areas in Lima. The asymmetrical distribution of sex is typical in psychology careers in Peru. All were single, without family responsibilities, and eventually had formal part-time jobs. The exclusion criteria were not consenting to participate and having produced an infrequent response pattern (see Results section).

**Table 3:** Sociodemographic summary of participants.

	N	%
Sex		
Male	29	23.2
Female	96	76.8
Preprofessional internship area		
Organizational–industrial	29	23.2
Social	6	4.8
Educational	23	18.4
Clinical	67	53.6

### Instruments

#### Coping strategy indicator–Short version (CSI-S^10^)

The CSI-S is an abbreviated measure of coping strategies (problem solving, support seeking and avoidance; PS, SS, and AVO, respectively) that was derived from the full Spanish version^42^; this indicator asks the respondent to write down in brief a problematic situation that is important to him/herself and then to answer 15 ordinally scaled items (*not at all*, *a little* and *a lot*; 5 items in each strategy). In the study of the adaptation, the reliability was satisfactory.

#### Single-item measure of stress (SIS^1^)

The SIS is a single-item measure that captures the experience of general stress based on emotional tension, restlessness and generalized worry, and was assessed with one item (Spanish translation: “*Estrés significa una situación en que una persona se siente tensa, inquieta, nerviosa o ansiosa, que tiene problemas para dormir, debido a su mente está preocupada todo el tiempo. ¿Usted se siente así en estos días?*”) scaled in five options (from ‘almost nothing’ to ‘a lot’). Due to its corroborated correlations with other variables, as an abbreviated measure, it represents an acceptable estimate of the overall stress experienced in several contexts^56,57^.

#### Efficacy to cope with difficulties (ECD)

The ECD item was developed as a proxy of the perceived global efficacy of coping with adversities experienced as difficulties. It was designed to represent the perceived effect on the ability to use coping strategies in the face of difficulties or stressors. It was phrased as “*Puedo hacer frente a las dificultades (problemas) que se me presentan*” (English translation: “I can cope with the difficulties (problems) that come my way"), scaled on five options (strongly disagree to strongly agree). This measure was influenced by the development of brief, efficient and valid measures applied in the work context^58^ and by global perceptions of effective coping that have been linked to constructs measured with the CSI-S.

### Procedure

#### Data collection

The study anticipated a relatively small sample size, but the sampling process aimed to ensure the voluntary participation of examinees. This anticipation also involved using nonparametric modeling (Mokken scaling analysis; see below), in which context at least 100 participants represented a reasonable sample size^48^. One author managed the academic coordination of the institutions with the goal of obtaining permission to administer the instruments during class time. The collection was carried out between January and April 2019; it was face-to-face, in groups of between 3 and 10 participants. The order of the material was constant (informed consent, sociodemographic sheet and instruments). The entire procedure was performed according to the Declaration of Helsinki, emphasizing the voluntariness of participation, anonymity of response, confidentiality of data, and freedom to discontinue participation at any point. There were no material incentives for participation.

#### Analysis

First, a nonparametric framework was used to establish the scaling properties of the CSI-S items. The dimensionality was examined using the Poly-DETECT algorithm for ordinal items^59^, with both confirmatory (to verify multidimensionality) and exploratory (to verify the number of dimensions) specifications. The following dimensionality indicators were used^60^: DETECT (> 1.0, strong multidimensionality), ASSI and RATIO (in both, a value of 1.0 indicates a dimensionally simple structure).

Second, the nonparametric item response theory model Mokken scaling analysis (MSA^61^) was used to (a) corroborate scaling (coefficient H ≥ 0.30^62^), (b) evaluate the model for monotonic homogeneity (cutoff for model acceptance, CRIT < 80), (c) explore local independence (with coefficients W^(1)^, W^(2)^ and W^(3)^^63^), and (d) obtain a measure of reliability of the observed scores^64^. To reduce the impact of irrelevant or invalid responses for MSA, we used the G^+^ coefficient^61^, which identifies participants with scores that violate the Guttman model. The R program *mokken*^61^ was used.

Third, another nonparametric item response theory approach, *Ramsay curves* (RC^65,66^), was used to obtain optimal scores constructed by kernel smoothing in the regression of the items and the ranking of the individuals derived from the direct score. Accordingly, regression weights were obtained to construct a maximum likelihood score (p_ML_) for each subject, which maximized the validity of the scaling of subjects on the observed score^43^. The R program *KernSmoothIRT*^67^ was used.

In the analysis for assessing the relation with external variables, ordinal regression was applied, with the p_ML_ scores of the PS, SS and AVO scales as predictors of efficacy to cope with difficulties (ECD) and single-item stress (SIS), with a flexible threshold model. For the single-item stress response, the logit link function was used due to the low frequency of response in the highest response category (n = 4, 3.2%). To reduce convergence problems and large standard errors^56^, option 4 (n = 14, 11.2%) and option 5 (n = 18, 14.4%) of the SIS items were combined with the adjacent category. For the ECD response, options 1 and 2 were combined, and the clog-log link function was used due to slight left skewness of their distribution. The R program *ordinal*^68^ was used.

### Ethics approval and consent to participate

The study was conducted in accordance with the guidelines of the Declaration of Helsinki and approved by the Commissions of Research, Ethics and Biosafety (Comisiones de Investigación, Ética y Bioseguridad), Hospital Infantil de México Federico Gómez, National Institute of Health. HIM/2015/017/SSA.1207, “Effects of mindfulness training on psychological distress and quality of life of the family caregiver”.

## Data Availability

The raw data used to support the conclusions of this article will be made available by the authors without undue reservation. Correspondence and requests for materials should be addressed to Filiberto Toledano-Toledano.
